# Effects of Nutrition, and Physical Activity Habits and Perceptions on Body Mass Index (BMI) in Children Aged 12–15 Years: A Cross-Sectional Study Comparing Boys and Girls

**DOI:** 10.3390/children8040277

**Published:** 2021-04-03

**Authors:** Vilelmine Carayanni, Elpis Vlachopapadopoulou, Dimitra Koutsouki, Gregory C. Bogdanis, Theodora Psaltopoulou, Yannis Manios, Feneli Karachaliou, Angelos Hatzakis, Stefanos Michalacos

**Affiliations:** 1 Laboratory of Statistical Modelling and Educational Technology in Public and Environmental Health–sepeh.lab, University of West Attica, 196 Alexandras Avenue, 11521 Athens, Greece; 2 Department of Endocrinology, Children’s Hospital P. & A. Kyriakou, Thivon & Levadeiasstr, Ampelokipoi T.K., 11527 Athens, Greece; elpis.vl@gmail.com (E.V.); fenkar1@hotmail.com (F.K.); stmichalakos@gmail.com (S.M.); 3 School of Physical Education & Sports Science, National and Kapodistrian University of Athens, 41, EthnikisAntistaseosstr, Daphne, 17237 Athens, Greece; dkoutsou@phed.uoa.gr (D.K.); gbogdanis@phed.uoa.gr (G.C.B.); 4 Department of Hygiene, Epidemiology and Medical Statistics, School of Medicine, National and Kapodistrian University of Athens, 75 MikrasAsias Str., 11527 Goudi, Greece; tpsaltop@med.uoa.gr (T.P.); ahatzak@med.uoa.gr (A.H.); 5 Department of Nutrition & Dietetics, School of Health Science & Education, Harokopio University, 70, El Venizelou Ave Kallithea, 17671 Athens, Greece; manios@hua.gr

**Keywords:** adolescents, nutrition habits, nutrition perceptions, Body Mass Index (BMI) category, overweight, obesity, physical activity habits, physical activity perceptions, Greece

## Abstract

*Background*: The aim of the present study was to examine the effects of socioeconomic status, nutrition and physical activity lifestyle habits and perceptions on Body Mass Index (BMI) in children aged 12–15 years in Greece. Furthermore, to compare the difference between the two sexes. *Methods:* This is a cross-sectional study conducted on a representative secondary school cohort that included 5144 subjects, aged 12 to 15 years. Students and their parents filled in validated questionnaires evaluating socioeconomic status, nutrition and physical activity. International Obesity Task Force cut offs were used to classify the children. Factor analysis of mixed data and partial proportional ordered logistic models were used to analyze ΒMΙ distributions. All analyses were stratified by gender. *Results:* Boys were 2.9 (95%CI: 2.592–3.328) times more likely to be overweight/obese than girls. Partial proportional ordinal models indicate significant associations between nutritional and physical habits and perceptions variables but also significant gender differences in socio-demographic, nutritional risk factors as well as physical activity habits and perceptions. *Conclusions:* A clear understanding of the factors that contribute to the sex differences in nutrition and physical activity habits and perceptions may guide intervention efforts.

## 1. Introduction

The prevalence of obesity during childhood and adolescence has risen significantly over the last decades, specifically according to NHANES study the rate of obesity during adolescence has quadrupled [1] The World Health Organization (WHO) has announced that childhood and adolescent obesity is the number one problem of public health and advises on actions needed to halt the progression of obesity epidemic [2].

Greece among other countries of southern Europe has one of the highest prevalence rates of childhood obesity [3]. Obesity is of multifactorial etiology including genetic, environmental such as nutrition and physical activity, and socioeconomic factors [4,5]. Obesity is associated with short-and long-term complications and comorbidities including metabolic syndrome, diabetes mellitus type 2 and premature cardiovascular disease [6,7].

In order to implement prevention strategies, it is very important to define the modifiable factors that contribute to the increase of body mass index (BMI). These factors can be recognized at an individual, family, community or national level [1,4,5]. In regards to sex there are several biological (sex specific) factors that have to do with the timing of puberty, body composition and growth patterns and social and cultural factors (gender specific) that reflect the ways in which boys and girls react to their physical and social environment [5]. Previous observational studies have examined associations of gender with behaviors linked to obesity and have observed gender related differences, namely that participation in vigorous exercise and in school sports had a protective effect for boys while drinking milk had a protective effect for girls [8]. It has also been reported that there are certain behaviors, especially eating disordered behaviors, as well as perceptions, such as fear of negative evaluation and weight shape concerns, that are gender dimorphic [9,10]. Furthermore, prevalence of components of metabolic syndrome and lifestyle behaviors may vary among the two sexes [11].

To our knowledge, there is limited information concerning the differences among boys and girls in adolescence, in regard to nutrition and physical habits and perceptions and their influence on BMI. The sex differences in acquired behaviors and perceptions vary across cultures and their impact on childhood obesity remains unclear. Previous research indicates that girls naturally require less energy intake than boys and that girls may be more attentive to food as well as to its effects on health and weight control [12].

The aim of the present study was to examine the effects of nutrition and physical activity lifestyle habits and perceptions on BMI in a large representative sample of children aged 12–15 years in Greece and to examine possible differences between the two sexes.

## 2. Materials and Methods

The “Hellenic Action Plan for the Assessment, Prevention and Treatment for Childhood Obesity” was a school-based survey, financed by the National Strategic Reference Framework, conducted at a national level in Greece and provided the data that were used in the present analysis. The study was conducted according to the guidelines of the Declaration of Helsinki, and approved by the Greek Ministry of National Education and the Greek Ministry of Health and the Hellenic Data Protection Authority (ethical code MIS 301205). Data were collected between January2015 and June 2015. The study population comprised schoolchildren attending secondary schools located in several municipalities in Greece (urban, semi-urban and rural areas, including islands). Semi-urban population includes the population of municipalities and communities, whose most populous settlement has 2000–9999 inhabitants, except those belonging to urban planning. Details for all regions and prefectures selected into the sample and their participation rate were given in Appendix A (Table A1).

Probability proportional to size (PPS) sampling was applied. The sampling of schools was stratified by region, proportionally to the total number of pupils attending these schools. Following this procedure, an appropriate number of schools were randomly selected from each one of these regions. Specifically, 73 secondary schools were included. Prior to signing an informed consent, an extended letter explaining the objectives of the study was provided to all parents or guardians whose children were attending these schools. Parents who approved participation of their children to the study proceeded to sign the informed consent form. Pupils with severe chronic illnesses i.e., malignancies, diabetes mellitus, rheumatoid arthritis or systemic lupus erythematosusor receiving chronic therapies for more than 6 months per year for any diagnosis, were excluded from the analysis (43 children).

The response rate was 72%, as 5180 parents out of the 7246 who signed parental consent forms have fully completed the questionnaire. After examination of univariate statistics to detect any anomalies in the distribution of variables, (especially outliers or missing values), aberrant values and duplicates (36 cases on the total), the total study sample in this analysis included 5144 secondary school children.

### 2.1. Measurement

Anthropometric measurements were conducted by sixteen health professionals, hired and trained for the purposes of this study. Children were measured by two trained members of the research team, using the same equipment and protocol. Body weight was measured to the nearest 100 g using a Tanita digital scale (Tanita BWB 800MA). Adolescents were weighed without shoes, with minimal possible clothing. Height was measured to the nearest 0.1 cm using a commercial stadiometer (Charder HM 200P Portstad). The Charderstadiometer was standardized against a Harpenden portable stadiometer. During the measurement of height, each adolescent was standing barefoot, keeping shoulders in a relaxed position, arms hanging freely, and head aligned to the Frankfort horizontal plane. Anthropometric measurements were taken three times, and the arithmetic mean of the three measurements was computed. Body weight and height were used one to calculate BMI, using the Quetelet’sequation, i.e., body weight (kg)/height^2^ (m^2^). BMI was calculated and subsequently adolescents were categorized according to International Obesity Task Force (IOTF) criteria [13] into the following four BMI categories: Underweight, Normal, Overweight and Obese. Both inter-rater and intra-rater reliability, measured with the intra-class correlation coefficients (ICC), yielded values greater than 0.97.

### 2.2. Questionnaires

Two structured questionnaires were developed and administered to the parents and students. Validity and reliability of the two questionnaires was tested and found to satisfy the principles of reliability [14]. In order to assess reliability, prior to initiation of the study, data were collected from 450 parents of children aged 12–15 and 250 adolescents aged 12–15 from the region of Attica. Cronbach’s alpha (α) and ICC were used to test reliability. Cronbach’s alpha and ICC showed acceptable reliability (α: 0.79 = 0.90 and ICC: >0.690–0.910). The factors addressed included socio-demographic characteristics, such as age, gender, geographic area (urban, semi urban, rural), nutrition and physical activity habits and perception, socioeconomic status (SES). SES index has a range between of 0–13, with higher values indicating higher SES of the family, as previously published by Moschonis et al., 2013 [15].

### 2.3. Statistical Analysis

Factor Analysis of Mixed Data

A mixed data factor analysis (FAMD) was attempted in order to reduce the number of variables. Factor Analysis of Mixed Data is a main component method dedicated to exploring data with both continuous and categorical variables. This factor analysis is suitable when the dataset includes both qualitative and quantitative variables. It can be seen roughly as a mix between Principal Component Analysis and Multiple Correspondence Analysis [16,17]. This ensures a balance between the influence of both continuous and categorical variables in the analysis.

The initial number of variables was 180. We have removed all items with a communality score less than 0.2 [18]. Items with low communality scores may indicate additional factors which could be explored in further studies by measuring additional items [19]. Any item which did not load above 0.3 on any factor was removed and the analysis was re-run. All retained factors had at least three items [20,21]. Items in the rotated factor matrix that cross-loaded on more than one factor were removed in turn, starting with the item with the highest ratio of loadings on the most variables with the lowest highest loading. Finally, 9 factors were retained with 32 variables that explain 59% of the total variance [22]. Results support the factorability of the correlation matrix.

**Figure A1:**
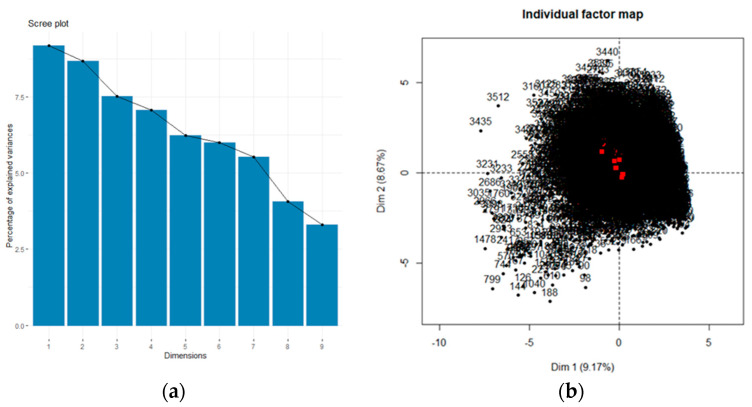
(**a**,**b**) Scree plot for each of the first 9 FAMD dimensions and individual factor map.

**Figure A2:**
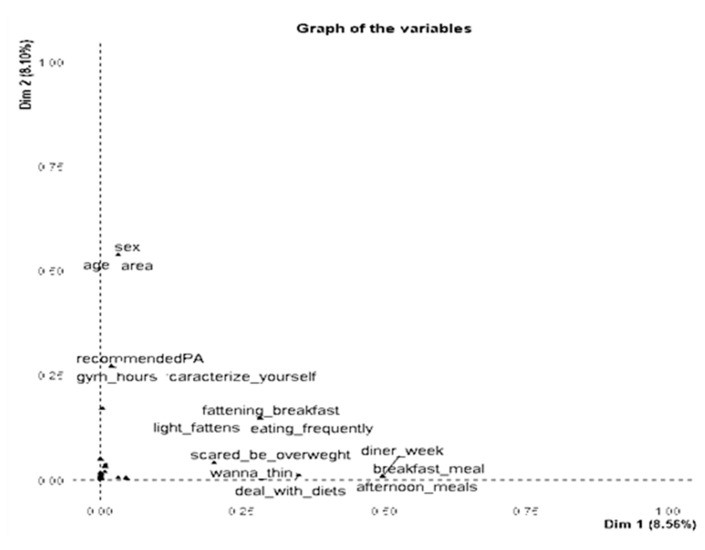
The first 15 well projected variables for the first two dimensions.

Figure A1a (screeplot) and Figure A1b in Appendix A indicate respectively the percentages of inertia explained by each of the final 9 FAMD and the individual factor map. Figure A2 indicates the first 15 well projected variables for the first two dimensions. As can be seen in Figure A2, eating habits (meals frequency) and fattening perceptions (five meals, skipping breakfast, eating frequently, eating light products), as well as socio-demographic characteristics (age, area, sex), have the most important contribution to the two first dimensions Variables related to body image (such as “I want to be thin” and “I deal with diets”), have the most important contribution to dimension 3. Perceptions for physical activity (such as self-evaluation on physical activity and efficiency of school’s gym hours and breaks), midday and after meal snacks (cheese pie, bread and fruits after dinner) and drinks in breakfast (such as cocoa milk, and whole milk), had respectively the most important contribution to the dimensions 4, 5 and 6). Organized sports (such as football, dance and track and field sports) had the most important contribution to Dimension 7. Purchase criteria/choice of food (according to their price or their calories) and SES, had the most important contribution to Dimension 8. Sedentary behaviors combined with eating such as meals in front of PC per week, snack (salty) in front of PC/tv per week, snack (sweet) in front of PC (TV) per week, had the most important contribution to Dimension 9. Also, sex has a significant contribution on Dimension 2, 5 and 7, that supports our decision to analyze the 2 sexes separately in order to avoid the possible additional complexity in our model. Factors description is presented in Appendix A, Table A2.

Descriptive Statistics-Outcome

Descriptive statistics were calculated for the remained variables. Chi-square tests and *t*-student tests were used to test the homogeneity among boys and girls for each variable at significance level α = 5%.

Weight category was measured on an ordinal scale: the codification of the outcome variable is: 0 = Normal weight/underweight, 1 = Overweight, 2 = Obese.

Ordered logistic models

For ordered dependent variables *Y*, the ordered logit model is often the natural choice. In ordinal logistic regression, the odds of being at category *j* or lower is:

$$\frac{P\left(Y\leq j\right)}{P(Y>j)};j=1,\ldots ,c-1;P\left(Y\leq c\right)=1$$

The ordered logit model, however, features a very restrictive model assumption, the assumption of proportional odds, that is often rejected in empirical research.

The proportional odds assumption in this study implies that the distances between different BMI levels are the same. The generalized ordered logit model differs from the standard ordinal or proportional odds model in that it relaxes the proportional-odds assumption. It allows that the predictors may have different effects on the odds that the response is above a cut off point, depending on how the outcomes are dichotomized. The Partial Proportional Odds (PPO) model is preferred to the generalized ordinal logistic regression if some predictor variables violate the assumption and their effects are estimated freely across different categories of the ordinal response variable [23,24,25].

Firstly, univariate ordinal logit model was fitted and the Brant test was performed to evaluate the parallel assumption. This Brant test is used to compare the coefficients of beta from c-1 binary logits and gives a list of variables violated the parallel assumption. Constraints were imposed on the variables where the parallel assumption was not violated [23].

The variables found, by use of univariate analysis, to be associated with the outcome variable at the *p* < 0.20 level, were included in the initial models to determine which factors were independent predictors of the outcome variable in the study subjects. All the ordinal logistic models were estimated via Maximum Likelihood Estimation (MLE) technique. ML estimates are values of the parameters having the ML of producing the observed sample [25]. The likelihood equations lead to the unknown parameters in a non-linear function. The ordinal logistic regression model was fitted to the observed responsesusing the maximum likelihood approach. In general, the method of maximum likelihood produces values of the observed probability values. According to the Brant test, parallel lines assumption was significantly violated for two variables in boys’ model and for four variables in girls’ model. Subsequently, PPO and generalized ordered logit model (GOLM) were fitted to the data. Finally, a comparison of the multivariate models was made [24].

The results were recorded as frequencies (N) and percentages, means and standard deviations (SD), odds ratios (OR) with 95% confidence intervals (CI), and *p* values. Hence, OR > 1 indicate that higher category values make it more likely that the respondent will be in a higher category than the current one, while OR < 1 indicate that higher category values on the predictor increase the likelihood of being in the current or a lower category.

All analyses were stratified by gender. R language (packages factorMiner, factorExtra) as well as Stata 14.0 software were used.

## 3. Results

### 3.1. Sample Characteristics

Sample characteristics followed by homogeneity tests, (α = 5%), between sexes are given on Table A3 in Appendix A for all variables included in final models. Results for the rest of variables are given on Table A4 in Appendix A). The prevalence of overweight and obesity was 24.8% and 6.8% respectively. Boys were 2.9 (95%CI: 2.592–3.328) times more likely to be overweight/obese than girls.

Statistically significant differences were observed among boys and girls in their self-evaluation in relation to physical activity with girls being significantly less active than boys (*p* < 0.001) and in relation to the perception regarding efficiency of school’s gym hours and breaks (*p* = 0.001). Also, boys participated more frequently than girls in outdoor sport activities such as track and field (*p* = 0.037). Girls seem to have a better level of knowledge in relation to eating habits, such as their opinion on “eating five meals per day” (*p* < 0.001), their agreement to “skipping breakfast is fattening” (*p* < 0.001) and the consumption of cheese pie at breakfast (*p* < 0.001). Significant differences were observed in fruit after dinner consumption that is more frequent in boys (*p* = 0.023). Results are in the same direction in cocoa milk consumption at breakfast (*p* < 0.001) and in frequency in dinner consumption (*p* < 0.001). Also, girls seem to have greater involvement with diets (*p* < 0.001) and to buy more frequently food products according to their calories (*p* < 0.001). It seems that boys are less concerned with their body image (“Ι’m scared to get overweight <0.001”, “I want to be thin”: *p* < 0.001).

### 3.2. Results of Partial Proportional Odds Models

As can be seen on Table A5 in Appendix B, Partial proportional odds (PPO) models presented better performance for both sexes. The model which represented the best fit according to Akaike’s Information Criterion (AIC) and Bayesian Information Criterion (BIC) is PPO model in both sexes as it has the smallest AIC and BIC and it is also more parsimonious. Results are presented and interpreted for the significant predictors in univariate analyses. The variables associated with the outcome at the 5% significance level were maintained in the final models Table A6 in Appendix B presents the data for underweight/normal weight children and compares them with data from children with overweight/obesity. Similarly, the second panel, (Table A7 in Appendix B), compares children with obesity with all other BMI categories.

#### 3.2.1. Partial Proportional Odds Models (PPOM): Boys

**Predictors that do not violate the parallel line assumption**

Holding the other predictors constant, the increase in frequency of cocoa milk consumption in breakfast by one unit was negatively associated with the odds (95% OR: 0.314, 95% CI: 0.125–0.792) to be in higher BMI categories, from underweight/normal weight to overweight/obese or from all other categories to obese)

Track and field sports seem to be a protective factor for obesity: Boys who regularly took part in track and field training for at least 2–3 times per week were 0.454 times (95%CI: 0.239–0.864) less likely to be in higher BMI categories, compared with boys who did not participate in track and field sports.

Dealing with diets was positively associated with the likelihood of being in a higher BMI category. Boys who never followed any diet were 0.203 (95% CI: 0.084–0.592) times less likely to move to higher categories in contrast to those who usually follow diets.

The increase of the frequency of afternoon meal per week by one unit decreased the odds to move from normal weight/underweight to higher BMI categories or from all other BMI categories to obesity by 0.203 times (95%CI: 0.088–0.468).

As compared to a boy who is always concerned with the desire to become thinner, a boy who was sometimes/rarely concerned with the desire to become thinner is 0.272 times (95% CI: 0.159–0.596) less likely to be in higher BMI categories. As opposed to a boy who was always concerned with the desire to become thinner, a boy who is never concerned with the desire to become thinner was 0.246 times (95% CI: 0.149–0.496) less likely to be in higher BMI categories, holding the other predictors constant.

As compared to a boy who is “always scared”, a boy who is “rarely scared” is 2.504 times more likely to be in overweight or obese status, (95% CI: 1.242–5.048). As compared to a boy who is “always scared”, a boy who is “never scared” was 2.070 times (95% CI: 1.118–3.831) more likely to be in overweight or obese status, holding the other predictors constant.

The subjective estimate of the level of physical activity had a significant effect among both binary models. A boy who characterized himself as quite/very active was 0.691 times (95% CI: 0.550–0.868) less likely to be in higher BMI categories as compared to a boy who did not characterize himself as quite/very active.

The daily consumption of cheese pie at brunch increased the odds to be in higher BMI categories by 4.465 times (95% CI: 1.567–10.724) in contrast to those who never or rarely ate cheese pie at breakfast.

A boy who agreed with the statement that “eating frequently without order fattens”, as compared to a boy who strongly disagreed/disagreed with this, was 0.508 (95% CI: 0.275–0.940) times less likely to be in higher BMI categories, from under-weight/normal weight to overweight/obese or from other categories to obese, holding all other factors constant.

Eating bread “sometimes” as compared to “never” decreased the odds to be in higher BMI categories by 0.453 times (95% CI: 0.274–0.747) in both BMI comparisons, holding the other predictors constant.

**Predictors which violated the parallel regression assumption**

A boy who agreed/strongly agreed with the perception that “light products fatten was 1.902 times (95% CI: 1.139–3.175) more likely to be in higher BMI categories from underweight/normal to overweight/obese as compared to a boy who did not agree/strongly disagree with this perception. The odds ratio became larger when the final binary model was used to compare obese with the other BMI categories. Moreover, a boy who strongly agreed/agreed with this perception in the final comparison (obese versus other categories) increased the odds to be in the higher BMI category by 3.815 (1.927–7.554) times as compared to a boy who did not agree/strongly disagree with this perception.

A boy who ate often (3–4 times per week) fruit after diner was by 0.440 times (95% CI: 0.195–0.988) less likely to be in a higher BMI category as compared to a boy who never ate fruit after diner. This predictor becomes significant only in the comparison among obese and lower BMI categories.

#### 3.2.2. Partial Proportional Odds Models (PPOM): Girls

**Predictors which do not violate the parallel line assumption**

A girl who characterized herself as quite/very active was 0.714 (95% CI: 0.582–0.877) times less likely to be in higher BMI categories as compared to a girl who did not characterize herself as active, from underweight/normal weight to overweight/obese or from other categories to obese, holding all other factors constant.

Also, higher values of the variable “dinner frequency” were related with lower values of BMI categories. In terms of odds ratio, the increase in frequency of this variable was negatively associated with the odds (OR: 0.863, 95% CI: 0.748–0.997) of being to a higher BMI category, from underweight/normal weight to overweight/obese or from other categories to obese, holding all other factors constant.

Increased frequency of eating bread (“often/always” as compared to “never”) at brunch, decreased the odds to be in higher BMI categories by 0.382 times (95% CI: (0.157–0.932), from underweight/normal weight to overweight/obese or from other categories to obese, holding all other factors constant.

Considering that five meals per day were good/very good (as oppossed to “bad/very bad”), decreases the odds to be in higher BMI categories by 0.241 times (96% OR: 0.079–0.735) from underweight/normal weight to overweight/obese or from other categories to obese, holding all other factors constant.

A girl who considered that the hours of physical activity in school classes and breaks are adequate in order to be physically active was 2.509 (95% CI: 1.338–4.704) times more likely to be in higher BMI categories as opposed to a girl who did not agree/strongly disagree with that.

Regular breakfast consumption seems to have a protective effect for obesity. In terms of odds ratio, the increase in frequency of this variable by one unit was negatively associated with the odds (OR: 0.537, 95% CI: 0.408–0.711) of being to a higher BMI category, from underweight/normal weight to overweight/obese or from other categories to obese, holding all other factors constant.

Considering that “skipping breakfast fattens” seems to have a protective effect against obesity. A girl who agreed that skipping breakfast consumption is fattenning as opposed to a girl who did not agree/strongly disagree with that, was 0.241 (95% CI:0.078–0.734) times less likely to belong to higher BMI categories, holding the other predictors constant.

A girl who was usually/often concerned with the desire to become thinner was 0.641 times (95% CI: 0.491–0.836) less likely to be classified in a higher BMI category as compared to a girl who was always concerned with this desire. Girls who are some-times or rarely concerned with the desire to become thinner were 0.476 times (95% CI: 0.351–0.646) less likely to be classified in a higher BMI category in comparison to girls who were always concerned with this desire. Also, girls who were never concerned with the desire to become thinner were 0.315 times (95% CI: 0.136–0.271) less likely to be classified in a higher BMI category when compared to girls who were always concerned with this desire.

Girls who were “scared of the idea of becoming obese” (from often through usually) were less likely to move to higher categories as compared to girls who are never “scared of the idea of becoming obese, from underweight/normal weight to overweight or from other categories to obesity”: As compared to a girl who is “always scared”, a girl who is “rarely scared” is 3.829 times more likely to be in overweight or obese status, (95% CI: 2.080–7.047). As compared to a girl who is “always scared”, a girl who is “never scared” is 2.438 times (95% CI: 1.305–4.354) more likely to be in overweight or obese status, holding the other predictors constant.

**Predictors which violated the parallel regression assumption**

Geographic area is a significant predictor only in the second binary model, when comparing obesity with other BMI categories. Girls who live in an urban area were 0.537 times (95% CI: (0.408–0.711)) less likely to be in the obesity category as opposed to girls who live in a rural area, holding the other predictors constant.

Age was negatively associated with the likelihood of being in a higher BMI category only when comparing underweight/normal weight with higher BMI categories. An age increase by 1 year, decreased the likelihood that a girl was classified in a higher BMI category by 0.668 times (95% CI: 0.584–0.813), holding the other predictors constant.

Girls who selected their foods according to their caloric content were 2.871 (95% CI: 1.042–7.901) times more likely to be in higher BMI categories than those who do not consider this criterion as important. This variable was significant, only in the final comparison (obesity versus other BMI categories).

Girls who drank whole milk at breakfast on a daily basis, were 2.483 (95% CI: 1.088–5.668) times more likely to be in obesity category than those who never drank whole milk at breakfast. This variable was significant, only in the final comparison (obesity versus other BMI categories).

## 4. Discussion

In the present study we addressed habits and beliefs that possibly lead to certain behaviors and choices in regards to eating and physical activity habits. It has been shown in previous research that a number of nutrition habits as well as sedentary activities are correlated with a positive increase of BMI [4]. Adolescents receive a large number of positive and negative messages from parents, teachers, caregivers and coaches regarding what consists a healthy lifestyle. Depending on their age, sex, intelligence, social and family environment they integrate in their beliefs a variable mixture of what they have been taught, and their thinking most likely reflects on their behavior (attitude). Boys and girls internalize stimuli in a different way and may react in distinct patterns.

In the representative sample of adolescents attending high schools throughout Greece, a number of differences among the two sexes were appreciated in regards to their habits, beliefs and fears concerning the factors that contribute to overweight and obesity. These beliefs had to do with what was more important in choosing the right food or avoid the unhealthy food i.e., calories, homemade or commercially available, the timing and number of meals during the day. The fear of obesity and the desire to lose weight or achieve a thinner body figure were also questioned. The subjective judgment of the level of activity and degree of physical fitness was addressed

The use of statistical tests and models has revealed the importance of a constellation of factors that correlate with higher BMI that are sex dimorphic. Girls seem to have greater involvement with diets and to buy more frequently food products according to their calories and are more concerned with their body image. Whilst avoidance of obesity is considered a healthy approach, inappropriate dieting and weight-loss behaviors known to accompany fear of fatness among adolescents, may pause a serious health risk [21]. It is possible that girls who have a desire to become thinner have a tendency to follow stricter diets for a short period of time and are at higher risk of emotional eating. Females are also more prone than boys to develop eating disorders [22].

Regarding their attitude towards dietary habits the notion that skipping breakfast is preventing for overweight was revealed to be a risk factor for obesity and the same applies for the concept that food choice according to their calorie content, is an important practice in order to halt BMI increase. It is anticipated that the attitude that skipping breakfast supports weight control is followed by omitting breakfast on a regular basis. The importance of breakfast intake on a daily base has emerged as an important factor preventing obesity, in several cross-sectional studies but not in cohort studies as it is published in a recent meta-analysis The risk of obesity in children and adolescents who skipped breakfast was 43% greater than those who ate breakfast regularly.

A possible explanation of the negative effect of food choices based on calorie content is that consumers frequently underestimate the caloric content of foods [26]. Another important factor is that it has been shown that paying attention only to caloric content does not assure the healthier choice as far as the content of trans-fat, carbohydrates, sugars, sodium and fiber that may not be optimal. Of interest is recent study that reports on the importance of the side where the label is placed and the impact that labeling has on consumer’s choice. They demonstrated that the label that has a positive statement such as healthy choice has stronger effect than a negative statement as high calorie food [27].

On the other hand a protective effect was elucidated for the impression that the consumption of five meals per day is a good practice. Girls who have the impression that consuming 5 meals per day, is a healthy practice have a higher possibility of applying this habit and also higher chance of having more family meals. Recent meta-analysis have demonstrated a significant relationship between frequent family meals and better nutritional health-in younger and older children, across countries and socioeconomic groups [28].It can also be contemplated that those girls make healthier choices of small meals or snacks and avoid frequent snacking or poor snack choices [29].

Referring to their judgment as to the adequacy of the time spent for physical activity in school as part of the physical education classes combined with the recreational activities and sports during school intermissions, a negative correlation was recognized. Adolescent girls who assume that this level of physical activity is appropriate as a daily standard of activity have higher chance of being in the higher level of BMI. This belief most likely leads to considerable inactivity during their leisure time during after school hours. Low levels of physical activity during adolescence are considered a global burden [30] and beliefs play an important role.

The subjective impression of the level of physical activity reflected to their BMI status as girls who considered themselves as being active or very active had lower chances to be in higher BMI categories than girls who do not characterize themselves as active. Again it is reasonable to contemplate that their self-judgment reflects on their involvement in sport activities.

Concerning their habits, girls who eat breakfast and dinner regularly have lower odds of obesity. To the same direction is the impact of consuming bread as part of brunch, especially for girls. This finding highlight the importance of eating breakfast as it was already stated but also the importance of having dinner in the context of having 5 meals per day.

Another factor that had a significant relation in a negative manner was the age. As girls move to higher class in high school had lower odds of being in the overweight/obese category. Specifically the increase of the age by 1 year decreases by 0.621 times the likelihood that a girl moves from underweight/normal weight to a higher BMI level. The impact of age can possibly be explained with the correlation that was noted that the desire to avoid overweight has a protective effect. As girls get older they have greater preoccupation with their body image and the fear obesity may play a stronger role.

In boys, concerning their beliefs and attitudes regarding what is healthy and what helps them to prevent overweight and obesity, it was elucidated that boys who think that unlimited snacking is related to a tendency to obesity were less likely to be overweight and obese. Meals and snacks are quite different in terms of way of preparation and setting in which they are consumed. It is most likely that these boys avoid an unordered way of eating and snacking throughout the day and thus attain a healthier body size. Recent study has shown that parents pay less attention and effort in preparing snacks as compared to meals and they are more prone to offer unhealthy snacks than unhealthy meals. Furthermore, they reported that this observation holds true for normal weight as well as overweight or obese children. In the same study differences were appreciated in the way snacks were treated among adolescent boys and girls with normal and increased BMI. It is notable that a greater percentage of non-overweight youth (both boys and girls) reported the consumption of fruits and vegetables as snacks, and boys, those of higher weight status were less likely than girls to eat foods prepared at home as snacks [31].

Boys who were preoccupied with diets and different types of dieting they were more likely to belong to the obesity group. This finding may be consistent with assumption that in order to lose weight or to maintain a healthy weight someone should follow a restrictive diet. This notion though may lead to a vicious cycle as restrictive diets have been associated with short term weight loss but long term aggravation of BMI. Furthermore, restrictive diets are not recommended during childhood or adolescence [32]. A study from Brazil reported that adolescents with poor self-image and higher BMI had a higher trend to follow restrictive diets, with no sex predilection [33].

The perception that light products fatten was proven to be a risk factor that increases the odds of being in the obese group. This viewpoint most likely prohibits them from including light products to their daily nutrition program and rather uses other foods that conceivably are more calories dense. To our knowledge there are no similar reports in the literature. Similarly, the daily consumption of whole milk in girls increases the odds to be to obesity category.

Regarding boys attitude towards “thinness”, results are in the same direction as in girls, although boys are less concerned with their body image than girls. Remarkable is that boys and girls who are indifferent of the status of obesity, “they do not have a fear of becoming obese”, have higher tendency to belong to the higher BMI group, whereas the opposite is true if they don’t have any desire of being thin. Recent study has revealed that adolescents have developed phobias as a result of being discriminated [34].

Self-assessment of the level of physical activity has a negative correlation with BMI category. The higher the impression of the level of physical activity the less the possibility to belong to the obesity group. This finding is in line with the observation in girls that underlines that adolescents can accurately estimate their level of activity and reflects their real-life level of activity.

Regarding boys’ nutritional habits several aspects are in line and support the importance of the having 5 meals a day in order to maintain a normal BMI. Boys who used to drink milk with cocoa for breakfast, have an afternoon snack, eat dinner, and/or have a fruit after dinner tend to be in the group of normal weight. It is of interest the finding that consumption of milk with cocoa has a protective effect. Similar findings for the protective effect of milk consumption in adolescent boys against obesity were reported by Murphy et al. [35]. The increased frequency of eating dinner was negatively associated with the odds of being in a higher BMI category. Eating bread for brunch or midday snack has a protective effect being a healthier choice compared with alternatives such as pies, cakes, and biscuits. On the other hand eating daily cheese pie for breakfast is a choice that increases the odds to be in the higher BMI group. This variable remains significant in the multivariate model for boys. Cheese pie is a high fat, high carbohydrate choice very popular among Greeks.

In regards to physical activity, boys who are involved in track and field sports had greater possibility of being in the normal weight group. Involvement in organized physical activity has been shown to be a protective affect for the development of obesity [36,37], however there is no clear impression as to what type of sport activity has a stronger effect. Track and field sports are more widely followed in Greece and this is reflected in the present study.

In regards to area of residence, living in a rural area remaιns significantly associated with obesity in the final models only for girls. The sociocultural differences, the differences in lifestyle habits and the lack of infrastructure in rural areas [36], that preclude the access of adolescents to gyms and sports facilities seems to have a more serious impact on the weight of girls than boys.

A limitation of the present study is the cross-sectional design which does not allow for causality conclusions and the self-reporting of information regarding sports participation and diet habits which is a source of bias. Longitudinal data are necessary to further unravel the complex interplay between the outcome and the above mentioned covariates. Strong points of the study are the large representative sample, the nationwide participation that increases the external validity of this study.

The current data support the hypothesis that beliefs and habits play an important and the perception role towards the development of obesity, however different factors may be protective as well as risk factors among the two genders. As was explicitly analyzed in the results section distinctive risk or protective factors emerged for the girls and boys. Regarding their impressions, the fear of obesity was protective for girls and for boys, whereas preoccupation with diets is a risk factor for obesity for both sexes. More healthy choices in mid-day snacks-such as bread-and frequent meals are protective factors for both sexes Gender dimorphic factors were recognized and these are the choice of food based on the calorie content for girls, frequent snacking for boys. Also, daily whole milk consumption at breakfast is associated with obesity in girls—although this consumption is more frequent for boys-, and high fat products consumption at breakfast that is more frequent for boys are related to increased risk for obesity for boys. On the other hand, regular cocoa milk consumption that is more frequent in boys than girls, seems to have a protective effect against obesity only for boys. Also girls seem to be more vulnerable to some sociodemographic factors such as age and geographic area. It is known that, beyond the biological differences and differences due to society or culture including food choices and dietary concerns, the levels of resting energy expenditure and energy requirements are different between girls and boys and the results of this study support these differences [38].

Regarding physical activity habits and perceptions, sex differences in engage in outdoor sport activities is evident in our study and this more frequent participation of boys emerges in this study as a protective factor against obesity as well [30,36].

A theoretical implication of these differences is that exposure to different role models, family dynamics as well as exposure to advertisements may affect the way children and adolescents appreciate weight status and factors correlating with weight control.

A practical implication is that the way doctors, parents and teachers approach children and adolescents promoting the healthy habits is of paramount importance as it can lead to negative or positive behaviors. An important finding of the present study is that the preoccupation with diets and dieting emerges as a risk factor for overweight and obesity. The tentative message is that positive role modeling and early education regarding healthy choices is desirable. Trying to understand more what the children think and criticize less could be helpful in helping them internalize health perceptions and follow healthy habits. The search for the perfect diet can have the opposite effect on BMI and this applies to both genders. Furthermore, the adherence to a five-mealplan per day has a protective effect and can prevent the development of obesity.

As public health measures are concerned, applying a prevention plan that includes psychological assessment and intervention taking into consideration the developmental differences of boys and girls as well as their perceptions which should be appreciated. Furthermore, ministry of education can implement measures for elementary schools on a regular basis and not as an intervention project of short duration taking into consideration the aspects of self-image, fear of obesity, preoccupation with dieting and diets. Having breakfast and mid-day snack at school together with the teachers routinely, will support the five meal plan per day.

Further research projects can address on a longitudinal basis, in prospective studies, the effect of alleviation of barriers on adoption of healthy life style habits considering the different needs of both sexes.

## 5. Conclusions

This study provides additional evidence that nutrition and physical activity habits and perceptions are important factors to prevent overweight/obesity in both sexes. Also, this analysis indicates that a number of perceptions and attitudes that correlate with obesity are sex dimorphic. A better understanding of trends in boys and girls nutrition habits and physical activity patterns within different weight status, seems to be a priority.

Interventions should be tailored according to sex in order to support adolescents to maintain healthy life style habits

## Data Availability

The data presented in this study are available on request from the corresponding author.

## Appendix A

**Table A1:** Regions and Prefectures included in the sample.

Regions	Prefectures	% of Sample
Eastern Macedonia Thrace	Drama	1.10
	Evros	1.10
	Kavala	1.10
	Xanthi	1.10
	Rodopi	1.10
Central Macedonia	Thessaloniki (2 regional units)	10.40
	Imathia	1.10
	Kilkis	1.10
	Pella	1.10
	Pieria	1.10
	Serres	2.20
	Halkidiki	1.10
Western Macedonia	Kastoria	1.10
	Kozani	2.20
	Florina	1.10
Epirus	Arta	1.10
	Thesprotia	1.10
	Ιoannina	2.20
	Preveza	1.10
Thessalia	Magnesia	2.00
	Larissa	2.70
	Trikala	1.50
Ionian Islands	Zakynthos	1.00
	Corfu	1.10
	Kefallinia	1.00
Western Greece	Aetoloakarnania	2.50
	Ahaia	3.20
	Viotia	1.80
	Evia	2.00
	Fthiotida	1.70
Attiki	Athens (6 regional units)	20.80
	Piraeus	6.30
Peloponnese	Argolida	1.10
	Arkadia	1.10
	Corinthοs	1.10
	Laconia	1.10
	Messenia	2.20
North Aegean	Lesvos	1.10
	Samos	1.10
	Chios	1.10
South Aegean	Cyclades (Samos, Santorini)	2.20
	Dodecanese (Rhodes, Kastelorizo)	2.20
Crete	Heraklion	2.60
	Lasithi	1.00
	Rethymno	1.00

**Table A2:** Factors extracted by Factor Analysis of Mixed Data and variables description.

Compounds	Eigenvalues	% Cumulative Variance	Description	Variable Labels	Variable Names
comp 1	3.85	9.167	Meals frequency and perceptions of fattening	Number of afternoon meals/week	afternoon_meals
				Frequency of dinner meals per week	diner_week
				Frequency of breakfast consumption per week	breakfast_meal
				Eating 5 meals per day is:	five_meals_per_day
				Eating frequently (without order) fattens	eating_frequently
				Skipping breakfast fattens	fattening_breakfast
				Eating light products fattens	light_fattens
comp 2	3.643	17.839	Sociodemographic factors	Age	age
				Area	area
				Sex	sex
Comp 3	3.155	25.45	Body images and behaviors against obesity and fattening	I want to be thin	wanna_be_thin
				I deal with diets	deal_with_diets
				I’m scared to be overweight	scared_be_overweght
Comp 4	2.969	32.55	Perceptions about physical activity	Self evaluation of physical activity	caracterize_yourself
				The recommended physical activity is 1 h	recommendedPA
				The gym hours and breaks at school are enough to be physically active	gym_hours
Comp 5	2.613	38.6	Foods/snacks eaten at meals and after meals	Bread at brunch	bread_brunch
				Cheese pie at breakfast	pie _ cheese
				Fruit after diner	fruit_afterdiner
Comp 6	2.517	44.6	Drinks consumed for breakfast	Whole milk at breakfast per week	whole_milk
				Cocoa milk at breakfast per week	cocoa_milk
				Fresh juice per week	freshjuice
Compound 7	2.319	50.31	Organized physical activity	Football per week	football
				Basket per week	basket
				Track and field per week	track_field
				Dance per week	danse_week
Comp 8	1.703	54.71	Purchase criteria/choice of food and socioeconomic status	Buying products according to their price	importance_of_low_cost
				Buying products according to their calories	count_calories
				ses	ses
comp 9	1.583	58.51	Sedentary behaviors and eating	Meals in front of PC per week	pc_food
				Snack (salty) in front of PC/tv per week	tv_snack
				Snack (sweet) in front of pc(TV) per week	pc_snack

**Table A3:** Descriptive statistics and statistical tests (α = 5%) by sex, for significant variables on multivariate models.

Variables (Codes)	Boys N = 2527 (49.00)	Girls N = 2617 (51.00)	*p*-Value ^1^
	N (%)	N (%)	
**BMI**			
Underweight/Normal Weight (0)	1574 (62.3)	1824 (69.7)	<0.001
Overweight (1)	661 (26.2)	574 (21.9)	
Obese (2)	209 (8.2)	130 (5.0)	
Missing	83 (3.3)	89 (3.4)	
**Area**			
Rural (0)	610 (24.1)	633 (24.2)	0.753
Semi urban (1)	462 (18.3)	492 (18.8)	
Urban (2)	1455 (57.6)	1492 (57.0)	
**Track and field per week**			
Never (0)	1699 (67.2)	1809 (69.1)	0.037
1 time (1)	319 (12.6)	271 (10.4)	
At least 2–3 times (2)	342 (13.6)	341 (13.0)	
Missing	167 (6.6)	196 (7.5)	
**Characterize yourself in relation to physical activity**			
No active/Not much either a little (1)	606 (24.0)	1067 (40.8)	<0.001
Quite/very active (2)	1921 (76.0)	1550 (59.2)	
**The gym hours and breaks at school are enough for me to be physically active**			
Strongly disagree/disagree (0)	1176 (46.5)	1300 (49.7)	0.001
Undecided (1)	559 (22.1)	628 (24.0)	
Agree/strongly agree (2)	740 (29.3)	647 (24.7)	
Missing	52 (2.1)	42 (0.6)	
**Eating cheese pie at breakfast**			
Νever/rarely (0)	1439 (56.9)	1764 (67.4)	<0.001
Sometimes/often (1)	847 (33.5)	629 (24.0)	
Daily (2)	68 (2.8)	29 (1.1)	
Missing	173 (6.8)	195 (7.5)	
**I deal with diets**			
Always (0)	64 (2.5)	111 (4.2)	<0.001
Sometimes/often (1)	243 (9.6)	337 (12.9)	
Rarely (2)	608 (24.1)	798 (30.5)	
Never (3)	1529 (60.5)	1299 (49.6)	
Missing	83 (3.3)	72 (2.8)	
**I am scared to get overweight**			
Always (0)	1065 (42.1)	1463 (55.9)	<0.001
Sometimes/often (1)	681 (26.9)	623 (23.8)	
Rarely (2)	440 (17.4)	345 (13.2)	
Never (3)	272 (10.9)	134 (5.1)	
Missing	69 (2.7)	52 (2.0)	
**I want to be thin**			
Always (0)	453 (17.9)	762 (29.1)	<0.001
Sometimes/often (1)	476 (18.8)	567 (21.7)	
Rarely (2)	634 (25.1)	610 (23.3)	
Never (3)	894 (35.4)	632 (24.1)	
Missing	70 (2.8)	46 (1.8)	
**Drinking whole milk at breakfast**			
Never/rarely	732 (29.0)	876 (33.5)	<0.001
Sometimes/Often	648 (25.6)	606 (23.2)	
Daily	933 (36.9)	912 (34.8)	
Missing	214 (8.5)	223 (8.5)	
**Eating bread at brunch**			
Never	635 (25.1)	643 (24.6)	0.079
Sometimes	1071 (42.4)	1201 (45.9)	
Often/always	629 (24.9)	607 (23.2)	
Missing	192 (7.6)	166 (6.3)	
**Eating fruit after dinner**			
Never	608 (24.0)	701 (26.8)	0.023
1–2 times	675 (26.7)	734 (28.0)	
3–4 times	553 (21.9)	507 (19.4)	
>4 times	623 (24.7)	610 (23.3)	
**Missing**	68 (2.7)	65 (2.5)	
**Buying products according to their calories**			
Not or little important	833 (33.0)	732 (28.0)	<0.001
Enough Important	726 (28.7)	610 (23.3)	
Very Important	858 (34.0)	1198 (45.8)	
Missing	110 (4.4)	77 (2.9)	
**Eating five meals per day is**			
Very bad/ bad	338 (13.4)	221 (8.4)	<0.001
Neither good or bad	560 (22.2)	519 (19.8)	
Good/very good	1540 (60.9)	1813 (69.4)	
Missing	89 (3.5)	64 (2.4)	
**Eating light products is fattening**			
Strongly disagree/disagree	1137 (45.0)	1220 (46.6)	0.574
Undecided	781 (30.9)	805 (30.8)	
Agree /Strongly agree	506 (20.0)	503 (19.2)	
Missing	103 (4.1)	89 (3.4)	
**Skipping breakfast is fattening**			
Strongly disagree/disagree	1501 (59.4)	1386 (53.0)	<0.001
Undecided	449 (17.8)	446 (17.0)	
Agree/strongly agree	485 (19.2)	722 (27.6)	
Missing	92 (3.6)	63 (2.4)	
**Frequency of breakfast consumption per week**	3.82 (1.37)	3.76 (1.43)	**0.102**
**Frequency of dinner meals per week**	4.03 (1.13)	3.80 (1.23)	**<0.001**
**Number of afternoon meals/week**	3.56 (1.26)	3.57 (1.28)	**0.839**
**Cocoa milk for breakfast per week**	3.82 (1.26)	1.71 (1.20)	**<0.001**
**Age**	13.55 (0.94)	13.54 (0.94)	**0.845**

^1^ The Chi-square test was used to calculate the *p*-value. Descriptive statistics and statistical tests (α = 5%) by sex (continuous variables). Bold: The *t*-student was used to calculate the *p*-value.

**Table A4:** Descriptive statistics and statistical tests (α = 5%) by sex for no significant variables on multivariate models.

Variables (Codes)	Boys N = 2527 (49.00)	Girls N = 2617 (51.00)	*p*-Value ^1^
	Boys	Girls	
The recommended physical activity is 1 h			
Strongly disagree/disagree (0)	356 (14.1)	221 (8.4)	<0.001
Undecided (1)	1256 (49.7)	1344 (51.4)	
Agree/strongly agree (2)	831 (32.9)	984 (37.6)	
Missing	84 (3.3)	68 (2.6)	
**Buying products according to their price**			
Not or little important (0)	1828 (72.3)	2154 (82.3)	<0.001
Enough Important (1)	401 (15.9)	255 (9.7)	
Very Important (2)	199 (7.9)	133 (5.1)	
Missing	99 (3.9)	75 (2.9)	
**Football per week**			
Never (0)	763 (30.1)	2027 (77.5)	<0.001
1 time (1)	328 (13.0)	361 (13.8)	
At least 2–3 times (2)	1414 (56.0)	219 (8.3)	
Missing	22 (0.9)	10 (0.4)	
**Basket per week**			
Never (0)	862 (34.1)	1445 (55.2)	<0.001
1 time (1)	600 (23.7)	429 (16.4)	
At least 2–3 times (2)	754 (29.9)	291 (11.1)	
Missing	311 (12.3)	452 (17.3)	
**Dance per week**			
Never (0)	1824 (72.2)	936 (35.8)	<0.001
1 time (1)	222 (8.8)	547 (20.9)	
At least 2–3 times (2)	86 (3.4)	768 (29.3)	
Missing	395 (15.6)	366 (14.0)	
Meals in front of PC per week	2.32 (1.087)	2.06 (1.066)	**0.015**
Snack (salty) in front of PC per week	1.990 (1.056)	1.910 (1.092)	**<0.001**
Snack (sweet) in front of TV per week	1.980 (1.079)	2.480 (1.054)	**<0.001**
SES	6.330 (1.841)	6.870 (0.900)	**<0.001**
Fresh juice per week	3.140 (1.315)	3.040 (1.310)	**0.008**

^1^ The Chi-square test was used to calculate the *p*-value. Bold: The *t*-student was used to calculate the *p*-value.

## Appendix B

**Table A5:** Log–likelihood and likelihood ratio estimates.

	Partial Proportional Odds Model PPOM		Generalized Ordered Logit Model GOLM	
	Boys	Girls	Boys	Girls
Log Likelihood (null)	−886.01	−708.56	−886.01	−708.56
Log Likelihood (model)	−599.02	−540.91	−585.40	−536.60
Likelihood Ratio chi2	573.96	335.38	580.60	339.44
*p*-value	<0.001	<0.001	<0.001	<0.001
Akaike’s Information Criterion	1442.06	1237.82	1455.30	1244.71
Bayesian Information Criterion	2046.29	1619.05	2064.45	1616.08

**Table A6:** Maximum likelihood estimates of Partial proportional odds model (overweight/obese versus other BMI categories).

Predictors	*p* > |z|		Odds Ratio (95% CI)	
Overweight/Obese Versus other BMI Categories				
**Characterize yourself in relation to physical activity**	Boys	Girls		
Quite/very active	0.001	0.001	0.691(0.550–0.868)	0.714(0.582–0.877)
**I think eating frequently (without order) is fattening**				
Undecided	0.845	NS ^1^	0.938 (0.294–1.166)	NS
Strongly agree / agree	0.031	NS	0.508 (0.275–0.940)	NS
**Eating bread for brunch**				
Sometimes/Rarely	0.002	0.065	0.453 (0.274–0.747)	0.468 (0.209–1.048)
Often/always	0.236	0.034	0.573 (0.228–1.439)	0.382 (0.157–0.932)
**Eating five meals per day is**				
Neither good or bad	0.493	0.232	1.198 (0.716–2.000)	1.376 (0.815–2.323)
Good/very good	0.384	0.012	1.231(0.771–1.966)	0.241(0.079–0.735)
**Area**	Boys	Girls	Boys	Girls
Semi-urban	0.972	0.293	1.009 (0.601–1.697)	0.767 (0.468–1.257)
Urban	0.327	0.387	0.816 (0.544–1.225)	0.837(0.560–1.252)
**Frequency of breakfast consumption per week**	0.373	<0.001	0.849 (0.359–2.011)	0.537 (0.408–0.711)
**Frequency of dinner meals per week**	0.089	0.045	0.955 (0.845–1.195)	0.863 (0.748–0.997)
**Eating cheese pie (breakfast)**				
Sometimes/often	0.402	0.072	1.204 (0.780–1.860)	0.810 (0.644–1.019)
Daily	0.005	0.081	4.465 (1.567–10.724)	0.319 (0.088–1.153)
**I am scared to get overweight**				
Sometimes/often	0.080	0.365	1.669 (0.941–2.959)	1.455 (0.890–2.379)
Rarely	0.010	<0.001	2.504 (1.242 5.048)	3.829 (2.080–7.047)
Never	0.021	0.005	2.070 (1.118–3.831)	2.438(1.305–4.554)
**I want to be thin**				
Usually/often	0.005	0.001	0.445 (0.251–0.788)	0.641(0.491–0.836)
Sometimes/rarely	<0.001	<0.001	0.272 (0.159–0.596)	0.476 (0.351–0.646)
Never	<0.001	<0.001	0.246 (0.149–0.496)	0.315 (0.136–0.271)
**Frequency of track and field per week**				
1 time per week	0.704	0.140	0.909(0.555–1.488)	0.669(0.392–1.141)
At least 2–3 times/week	0.016	0.437	0.454(0.239–0.864)	0.777 (0.412–1.468)
**Cocoa milk for breakfast per week**	0.014	NS	0.314 (0.125–0.792)	NS
**I deal with diets**				
Sometimes/often	0.581	0.878	0.718(0.22198–2.325)	1.071 (0.444–2.582)
Rarely	0.858	0.551	0.901 (0.288–2.819)	1.324(0.526–3.331)
Never	0.018	0.377	0.258(0.084–0.592)	1.324(0.526–3.331)
**Number of afternoon meals/week**	<0.001	0.311 (0.809–1.947)	0.203 (0.088–0.468)	1.255(0.809–1.947)
**Eating light products is fattening**				
Undecided	0.392	NS	1.307 (0.708–2.415)	NS
Agree /Strongly agree	<0.001	NS	1.902 (1.139–3.175)	NS
**Skipping breakfast is fattening**				
Undecided	0.384	0.013	1.231 (0.771–1.966)	1.376 (0.815–2.323)
Agree/strongly agree	0.493	0.232	1.197(0.716–2.000)	0.241(0.078–0.734)
**The gym hours and breaks at school are enough for me to be physically active**				
Undecided	ΝS	0.121	ΝS	0.352 (0.699–2.154)
Agree/strongly agree	ΝS	0.004	ΝS	2.509(1.338–4.704)
**Age**		<0.001	NS	0.668(0.548–0.813)
**Drinking whole milk at breakfast**				
Sometimes/often	0.244	0.467	0.394 (0.798–2.422)	0.895 (0.508–1.578)
Daily	0.247	0.187	0.725 (0.451–1.168)	0.765 (0.486–1.204)
**Buying products according to their calories**				
Enough Important	NS	0.041	NS	2.871 (1.042–7.901)
Very Important	NS	0.810	NS	0.926 (0.495–1.731)
**Eating fruit after dinner**				
1–2 times	0.560	0.712	0.919 (0.585–1.441)	1.151 (0.717–1.849)
3–4 times	0.803	0.997	1.001 (0.616–1.626)	1.071 (0.625–1.836)
>4 times	0.352	0.232	0.679(0.360–1.281)	0.727 (0.539–1.848)

^1^ Non Significant in univariate analysis.

**Table A7:** Maximum likelihood estimates of Partial proportional odds model (obese versus other BMI categories).

Predictors	*p* > |z|		Odds Ratio (95% CI)	
Obese Versus other BMI Categories				
**Characterize yourself in relation to physical activity**				
Quite/very active	0.001	0.004	0.691 (0.550–0.868)	0.714 (0.582–0.877)
**I think eating frequently (without order) is fattening**				
Undecided	0.845	NS	0.938 (0.294–1.166)	NS
Strongly agree/agree	0.031	NS	0.508 (0.275–0.940)	NS
**Eating bread for brunch**				
Sometimes	0.002	0.065	0.453 (0.274–0.747)	0.468 (0.209–1.048)
Often/always	0.236	0.034	0.573 (0.228–1.439)	0.382 (0.157–0.932)
**Eating five meals per day is**				
Neither good or bad	0.493	0.232	1.198 (0.716–2.000)	1.376 (0.815–2.323)
Good/very good	0.384	0.012	1.231 (0.771–1.966)	0.241 (0.079–0.735)
**Area**	Boys	Girls	Boys	Girls
Semi-urban	0.972	0.293	1.009 (0.601–1.697)	0.767 (0.468–1.257)
Urban	0.327	0.005	0.816 (0.544–1.225)	0.332 (0.154–0.712)
**Frequency of breakfast consumption per week**	0.373	<0.001	0.849 (0.359–2.011)	0.537 (0.408–0.711)
**Frequency of dinner meals per week**	0.089	0.045	0.955 (0.845–1.195)	0.863 (0.748–0.997)
**Eating cheese pie (breakfast)**				
Sometimes/often	0.402	0.072	1.204 (0.780–1.860)	0.810 (0.644–1.019)
Daily	0.005	0.081	4.465 (1.567–10.724)	0.319 (0.088–1.153)
**I am scared to get overweight**				
sometimes/often	0.080	0.365	1.669 (0.941–2.959)	1.455 (0.890–2.379)
rarely	0.010	<0.001	2.504 (1.242–5.048)	3.829 (2.080–7.047)
Never	0.021	0.005	2.070 (1.118–3.831)	2.438 (1.305–4.554)
**I want to be thin**				
Usually/often	0.005	0.001	0.445 (0.251–0.788)	0.641 (0.491–0.836)
Sometimes/rarely	<0.001	<0.001	0.272 (0.149–0.496)	0.476 (0.351–0.646)
Never	<0.001	<0.001	0.246 (0.149–0.496)	0.315 (0.136–0.271)
**Frequency of track and field per week**				
1 time per week	0.704	0.140	0.909 (0.555–1.488)	0.669 (0.392–1.141)
At least 2–3 times/week	0.016	0.437	0.454 (0.239–0.864)	0.777 (0.412–1.468)
**Cocoa milk for breakfast per week**	0.014	NS	0.314 (0.125–0.792)	NS
**I deal with diets**				
Sometimes/often	0.581	0.878	0.718 (0.22198–2.325)	1.071 (0.444–2.582)
Rarely	0.858	0.551	0.901 (0.288–2.819)	1.324 (0.526–3.331)
Never	0.018	0.377	0.258 (0.084–0.792)	1.324 (0.526–3.331)
**Number of afternoon meals/week**	<0.001	0.311 (0.809–1.947)	0.203 (0.088–0.468)	1.255 (0.809–1.947)
**Eating light products is fattening**				
Undecided	0.392	NS	1.307 (0.708–2.415)	NS
Agree /Strongly agree	<0.001	NS	3.815 (1.927–7.554)	NS
**The gym hours and breaks at school are enough for me to be physically active**				
Undecided	ΝS	0.121	ΝS	0.352 (0.699–2.154)
Agree/strongly agree	ΝS	0.004	ΝS	2.509 (1.338–4.704)
**Drinking whole milk at breakfast**				
Sometimes/often	0.244	0.467	0.394 (0.798–2.422)	0.895 (0.508–1.578)
Daily	0.247	0.031	0.725 (0.451–1.168)	2.483 (1.088–5.668)
Age	NS	0.861	NS	0.967 (0.663–1.410)
**Buying products according to their calories**				
Enough Important	NS	0.203	NS	1.478 (0.810–2.697)
Very Important	NS	0.810	NS	0.926 (0.495–1.731)
**Eating fruit after dinner**				
1–2 times	0.560	0.712	0.919 (0.585–1.441)	1.151 (0.717–1.849)
3–4 times	0.047	0.997	0.440 (0.196–0.988)	1.071 (0.625–1.836)
>4 times	0.352	0.232	0.679 (0.360–1.281)	0.727 (0.539–1.848)
